# Dynamic Survival Patterns in Rural-Urban Health Disparities Among Chinese Older People

**DOI:** 10.1093/geroni/igaf122.3419

**Published:** 2025-12-31

**Authors:** Shibin Yan

**Affiliations:** University of Pennsylvania, Philadelphia, Pennsylvania, United States

## Abstract

China’s rapidly aging population faces significant rural-urban health disparities, yet limited research has applied survival analysis to assess their impact on longevity. This study investigates how rural-urban disparities influence life expectancy among Chinese older adults (aged 65+) using data from the Chinese Longitudinal Healthy Longevity Survey (CLHLS, 2005–2010). Survival analysis, including Kaplan-Meier estimates and Cox proportional hazards models, was employed to compare survival experiences and identify predictors of mortality. The sample comprised 7,472 older adults (3,408 urban, 4,064 rural). Results revealed dynamic survival patterns: urban residents exhibited higher survival probabilities between years 10–13, but rural older adults showed marginally better survival after 14 years. Cox regression found urban residence associated with a 10.7% higher mortality hazard (HR = 1.11, p = 0.01), contrary to initial hypotheses (H1: significant rural-urban disparities; H2: urban advantage in longevity). Key predictors of longevity included age, financial sufficiency (HR = 0.86, p < 0.001), female gender (HR = 0.80, p < 0.001), and better self-rated health (HR = 0.94, p = 0.004). Social networks in rural areas may partly explain later-life survival advantages. The study highlights the complex role of geographic disparities and socioeconomic factors in shaping longevity. Findings underscore the need for policies addressing structural inequities in healthcare access while leveraging rural social capital to improve older adults’ well-being. This research contributes novel insights by applying survival analysis to rural-urban health disparities in China, emphasizing temporal variations in mortality risks and advocating for targeted interventions to reduce health inequalities.

